# Mechanism of Antifungal Activity by 5-Aminoimidazole-4-Carbohydrazonamide Derivatives against *Candida albicans* and *Candida krusei*

**DOI:** 10.3390/antibiotics10020183

**Published:** 2021-02-12

**Authors:** Fátima Cerqueira, Marta Maia, Carla Gabriel, Rui Medeiros, Sara Cravo, Ana Isabel Ribeiro, Daniela Dantas, Alice Maria Dias, Lucília Saraiva, Liliana Raimundo, Eugénia Pinto

**Affiliations:** 1 Health Sciences Faculty, University Fernando Pessoa, 4200-150 Porto, Portugal; fatimaf@ufp.edu.pt (F.C.); carlapatriciagabriel@gmail.com (C.G.); ruimedei@ipoporto.min-saude.pt (R.M.); 2 FP-ENAS/CEBIMED, Energy, Environment and Health Research Unit/Biomedical Research Center, University Fernando Pessoa, 4249-004 Porto, Portugal; 3 Interdisciplinary Centre of Marine and Environmental Research (CIIMAR/CIMAR), University of Porto, 4450-208 Matosinhos, Portugal; scravo@ff.up.pt; 4 Laboratory of Microbiology, Biological Sciences Department, Faculty of Pharmacy of University of Porto, 4050-313 Porto, Portugal; martaabreumaia@gmail.com; 5 Molecular Oncology and Viral Pathology Group, Portuguese Oncology Institute (IPO), 4200-072 Porto, Portugal; 6 Laboratory of Organic and Pharmaceutical Chemistry, Department of Chemical Sciences, Faculty of Pharmacy, University of Porto, 4050-313 Porto, Portugal; 7 Centre of Chemistry, Department of Chemistry, University of Minho, 4710-057 Braga, Portugal; a.isabel.f.ribeiro@gmail.com (A.I.R.); dantasdaniela97@gmail.com (D.D.); ad@quimica.uminho.pt (A.M.D.); 8 Centre for Textile Science and Technology (2C2T), University of Minho, 4800-058 Guimarães, Portugal; 9 LAQV/REQUIMTE, Laboratory of Microbiology, Biological Sciences Department, Faculty of Pharmacy of University of Porto, 4050-313 Porto, Portugal; lucilia.saraiva@ff.up.pt (L.S.); liliana.s.g.raimundo@gmail.com (L.R.)

**Keywords:** *Candida* sp., antifungals, (*Z*)-5-amino-*N*’-aryl-1-methyl-1*H*-imidazole-4-carbohydrazonamides, mechanisms of action, reactive oxygen species, ergosterol, dimorphic transition, metabolic viability

## Abstract

Systemic mycoses are one major cause of morbidity/mortality among immunocompromised/debilitated individuals. Studying the mechanism of action is a strategy to develop safer/potent antifungals, warning resistance emergence. The major goal of this study was to elucidate the mechanism of action of three (*Z*)-5-amino-*N*’-aryl-1-methyl-1*H*-imidazole-4-carbohydrazonamides (2h, 2k, 2l) that had previously demonstrated strong antifungal activity against *Candida krusei* and *C. albicans* ATCC strains. Activity was confirmed against clinical isolates, susceptible or resistant to fluconazole by broth microdilution assay. Ergosterol content (HPLC-DAD), mitochondrial dehydrogenase activity (MTT), reactive oxygen species (ROS) generation (flow cytometry), germ tube inhibition and drug interaction were evaluated. None of the compounds inhibited ergosterol synthesis. Ascorbic acid reduced the antifungal effect of compounds and significantly decreased ROS production. The metabolic viability of *C. krusei* was significantly reduced for values of 2MIC. Compounds 2h and 2k caused a significant increase in ROS production for MIC values while for 2l a significant increase was only observed for concentrations above MIC. ROS production seems to be involved in antifungal activity and the higher activity against *C. krusei* versus *C. albicans* may be related to their unequal sensitivity to different ROS. No synergism with fluconazole or amphotericin was observed, but the association of 2h with fluconazole might be valuable due to the significant inhibition of the dimorphic transition, a *C. albicans* virulence mechanism.

## 1. Introduction

Systemic mycoses are one major cause of morbidity and mortality among immunocompromised (HIV infection, neutropenic and transplanted) and debilitated individuals attending health care institutions (e.g., intensive care units) [1,2]. *Candida* sp. are responsible for the majority of the yeast-like systemic mycosis [1,2]. In the last few decades, the increment of fungal infections, particularly nosocomial, and resistance to antifungal agents has stimulated the search for new antifungal agents [3,4].

The search for new antifungal drugs can be based on two different approaches: (i) the search for molecules with antifungal activity correlated with the interference in a specific target on the fungal cell and/or (ii) the search for molecules that can improve the activity of an already recognized antifungal agent [5]. The study of antifungals’ mechanisms of action constitutes an essential strategy to develop safer and potent compounds, as well as warning of the actual emergence of resistance to antifungal agents.

The main mechanisms of action described for commercial antifungal drugs are the interference with cell membrane (synthesis inhibition or interference with ergosterol, responsible for preserving cell integrity, viability, function and normal growth) and cell wall (synthesis inhibition of glucans) [5,6]. Besides the main effect on fungal cells, additional mechanisms of action that increase the antifungal activity of drugs include the inhibition of fungal virulence factors (as dimorphic transition, adhesion to host cells or biofilm formation) [7], induction of ROS production [5], interference with mitochondria activity or modulation of immune responses [8,9,10].

From all the classes of antifungals, azoles are the most widely used in antifungal therapy due to their broad spectrum, efficacy and clinical safety [6]. The antifungal activity of azole compounds, such as fluconazole, is mainly attributed to inhibition of 14*α*-demethylase leading to depletion of ergosterol with implications for the plasma membrane structure and function [11]. Nevertheless, besides this, other mechanisms as antifungal activity mediated by reactive oxygen species (ROS) have been put forward [12,13,14,15].

**Figure 1:**
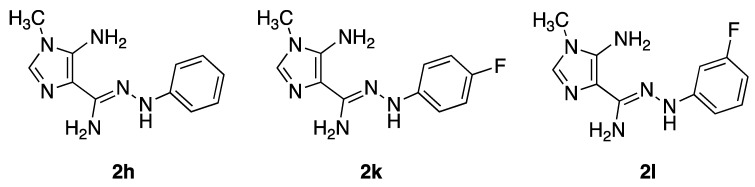
Lead antifungals: (*Z*)-5-amino-*N*’-aryl-1-methyl-1*H*-imidazole-4 carbohydrazonamide derivatives.

Our group had previously identified three (*Z*)-5-amino-*N*’-aryl-1-methyl-1*H*-imidazole-4-carbohydrazonamides (aryl = phenyl (2h), 4-fluorophenyl (2k), 3-fluorophenyl (2l)) as lead antifungal compounds (Figure 1) for *Candida* sp. infections [16]. Their effect on *C. albicans* and *C. krusei* biofilms on nanohydroxyapatite substrate was also previously reported [17].

The goal of the present work was to elucidate the mechanism of action underlying the antifungal activity of 5-aminoimidazole-4-carbohydrazonamide derivatives against *C. albicans* and *C. krusei*.

## 2. Results

### 2.1. Antifungal Susceptibility to Imidazole Derivatives

Minimal inhibitory concentration (MIC) and minimal lethal concentration (MLC) of imidazole derivatives and fluconazole were determined for *C. albicans*, *C. dubliniensis* and *C. krusei* strains and the results are shown in Table 1.

These results are in accordance with those previously described [16], with compound 2l possessing the higher antifungal activity among the three derivatives tested, and *C. krusei* growth being the most affected for the three compounds, when compared with the other *Candida* species.

For compound 2h, no difference was observed for the susceptibility of *C. albicans* strains tested (either for MIC or MLC), independently of their susceptibility to fluconazole. However, for compounds 2k and 2l, strain M1 was more susceptible than ATCC (American Type Culture Collection) and H37 strains. *Candida albicans* H37 is a fluconazole resistant strain, but was inhibited for all the compounds with equal concentrations as those necessary to inhibit *C. albicans* ATCC 10231 and the clinical isolate M1, both fluconazole susceptible. *Candida dubliniensis* had an analogous behavior to *C. albicans* when exposed to compound 2h (MIC 64 µg/mL). For compounds 2k and 2l, MIC values for *C. albicans* strains and *C. dubliniensis* ranged from 16 to 32 µg/mL and 8 to 16 µg/mL, respectively.

For *C. albicans* strains and *C. dubliniensis*, a fungistatic effect was achieved for compounds MIC values. On the other hand, for *C. krusei*, an intrinsically fluconazole resistant species, the activity is higher and the effect was fungicidal for the MIC values, considering compounds 2h and 2l.

Since the MIC values for compound 2h were defined as an interval, from here, for experiments testing interference with yeast functions, MIC values will refer to the higher concentration determined.

### 2.2. Interference with Ergosterol Synthesis

The interference of 5-aminoimidazole-4-carbohydrazonamide derivatives with ergosterol synthesis, the main mechanism of action described for azoles, was evaluated. The new imidazole derivatives tested did not reduce the ergosterol content on yeast cell membranes of *C. albicans* ATCC 10231 and *C. krusei* ATCC 6258, at sub-inhibitory concentrations and when compared with fluconazole (data not shown).

### 2.3. Antioxidants’ Influence on Antifungal Activity

Reactive oxygen species production is one of the mechanisms of antifungal action described to azole compounds. To determine the influence of the ascorbic acid (AA) on the activity of the 5-aminoimidazole-4-carbohydrazonamide derivatives, MICs were determined in the presence of AA (Table 2).

Ascorbic acid (5mM) annuls the inhibitory effect of the compounds 2h/2k/2l to at least a concentration four times higher than the MIC. When evaluated separately, at 5 mM, AA does not affect either *C. albicans* or *C. krusei* growth.

### 2.4. Effect on Cell Mitochondrial Function

**Figure 2:**
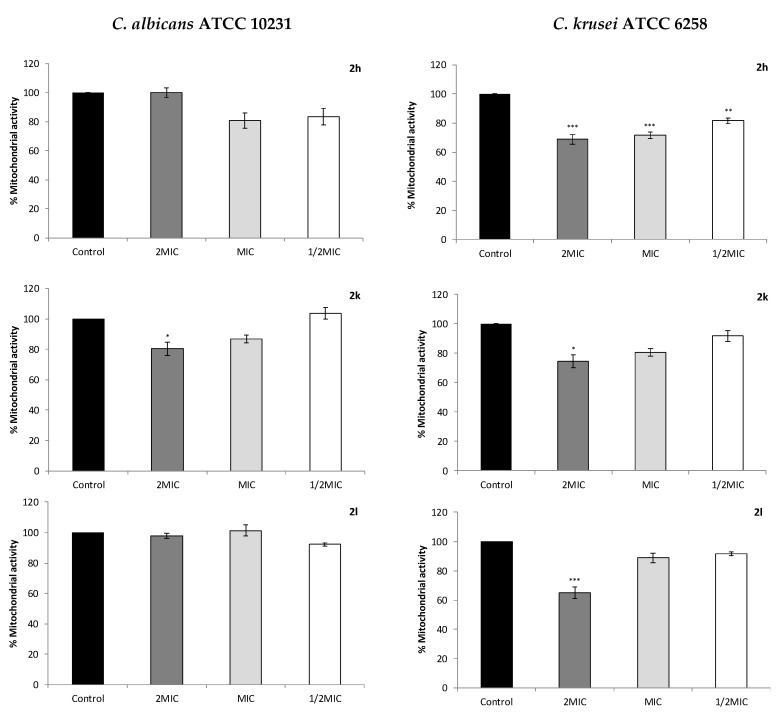
Mitochondrial activity of *Candida albicans* ATCC 10231 and *Candida krusei* ATCC 6258 cells treated with different concentrations of 5-aminoimidazole-4-carbohydrazonamide derivatives. Results are expressed as the means ± standard error of the mean (SEM) of three independent experiments. Controls included 1% of DMSO. MIC, minimal inhibitory concentration. * *p* < 0.05; ** *p* < 0.01; *** *p* < 0.001 (one-way ANOVA followed by Dunnett’s test).

To evaluate a possible interference of the imidazole derivatives with mitochondrial function of both *C. albicans* and *C. krusei*, the MTT reduction assay was performed (Figure 2). All the compounds were able to inhibit the mitochondrial viability of *C. krusei*; compound 2k also inhibited *C. albicans* mitochondrial viability for twice the MIC concentration (2MIC). For compound 2h a significant inhibition of the respiratory chain function was observed for *C. krusei*, even at half the MIC concentration (½MIC; 4 µg/mL), while for 2k and 2l a significant inhibition was only observed for the concentration of 2MIC.

For fluconazole, even at concentration of 2MIC, no inhibition of the mitochondrial activity was observed, the mitochondrial activity being equal or higher than the control for both *C. albicans* and *C. krusei*.

### 2.5. Effect on Total Intracellular ROS Production

**Figure 3:**
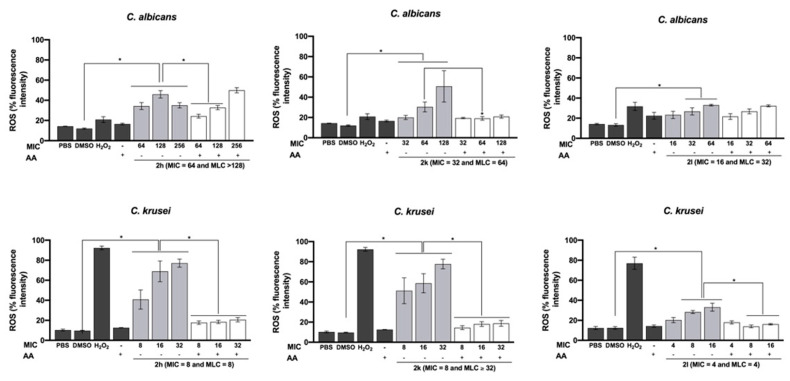
Effect of 5-aminoimidazole-4-carbohydrazonamide derivatives on reactive oxygen species (ROS) production in *Candida albicans* ATCC 10231 and *Candida krusei* ATCC 6258, after 6 h treatment. MIC, minimal inhibitory concentration. Ascorbic acid (AA; 5 mM) was used as antioxidant and H_2_O_2_ (1 mM) as positive control for ROS detection. +, with AA and -, without AA. Data are mean ± SEM, *n* = 2–3 independent experiments; values significantly different from DMSO (0.5%): * *p* < 0.05, (two-way ANOVA followed by Dunnett’s test).

As ROS production seemed to emerge as a possible mechanism for the 5-aminoimidazole-4-carbohydrazonamides antifungal activity, the total content of intracellular ROS was quantified using 2’,7’-dichlorodihydrofluorescein diacetate (H2DCFDA), which is indicated not only for the detection of H_2_O_2_ but also hydroxyl radical, hydroperoxides and peroxynitrite [18]. Moreover, since AA decreased the antifungal activity of the imidazole derivatives, their effect on ROS production by *C. albicans* and *C. krusei* was also evaluated (Figure 3). Imidazole derivatives (2h, 2k and 2l) stimulated ROS generation. Compounds 2h and 2k caused a significant increase in ROS production for all the concentrations tested, while 2l only caused a significant increase in ROS production for concentrations of 2MIC and 4MIC. The simultaneous incubation of yeasts with the imidazole derivative and AA significantly decreased intracellular ROS detection, except for 2l at MIC concentration.

### 2.6. C. albicans Virulence Mechanisms: Interference with Germ Tube Formation

**Figure 4:**
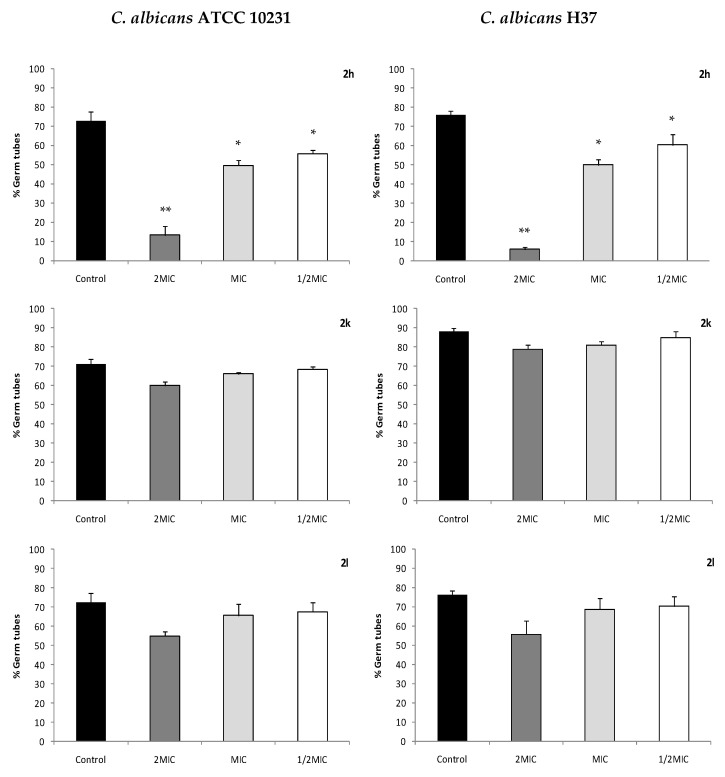
Percentage of germ tube formation by two *Candida albicans* strains (ATCC 10231 and H37) with different concentrations of 5-aminoimidazole-4-carbohydrazonamides derivatives. Results show means ± SEM of three independent experiments. Controls included 1% of DMSO. MIC: minimal inhibitory concentration. * *p* < 0.05; ** *p* < 0.001 (one-way ANOVA followed by Dunnett’s test).

To study the influence of imidazole derivatives on virulence factors of *C. albicans*, the effect of the compounds on *C. albicans* dimorphic transition was evaluated (Figure 4). Compound 2h caused a significant inhibition of the germ tube formation of the two *C. albicans* strains (ATCC and H37), for all the concentrations tested including 1/2MIC. For 2k and 2l compounds, no significant inhibition of germ tube formation was observed, even for the concentrations of 2MIC. For fluconazole, only a slight inhibition (2.4% to 8.3%) was observed for concentration of 2MIC.

### 2.7. Interaction Test by Checkboard Microdilution Assay

Imidazole derivatives were tested in blend experiments with the antifungals fluconazole and amphotericin B to evaluate whether there was a synergistic effect between these compounds. After determining the FICI (data not shown) no synergistic or antagonistic action was observed for the tested strains (FICI >0.5 and ≤4).

## 3. Discussion

*Candida krusei*, an intrinsic fluconazole resistant *Candida* species that was recently considered a potential multidrug-resistant yeast [19], along with some *C. albicans* multidrug-resistant strains, are challenging the scientific community for the search of new therapeutic molecules. The 5-aminoimidazole-4-carboxamidrazone derivatives tested are more potent against *C. krusei*, an advantage for their possible use in therapeutics [16], along with their activity against *C. dubliniensis* (*C. albicans* phenotypically similar) and *C. albicans* fluconazole sensitive and resistant strains. Besides that, compounds 2h and 2l are fungicidal for *C. krusei* for MIC concentrations.

Antifungal compounds with different and multiple mechanisms of action are currently used in therapeutics. Considering azoles, the principal family of antifungal compounds used in therapeutics, the main mechanism of action described is the inhibition of ergosterol synthesis. Ergosterol serves as a bioregulator of membrane fluidity and, consequently, is responsible for the membrane integrity of fungal cells [11]. When comparing imidazole derivatives (2h, 2k, 2l) effect on the growth of *C. albicans* containing ergosterol in the cell membrane (ATCC and M1 strains) with that of *C. albicans* H37 without ergosterol (named VSY2 in Vale-Silva et al., 2012) a similarity in MIC values was verified [20]. These results infer that the compounds do not act at the level of ergosterol. To confirm this hypothesis, the effect of the imidazole derivatives was tested against ergosterol synthesis. The content of ergosterol after imidazole treatment was not reduced, leading to the conclusion that these 5-aminoimidazole-4-carbohydrazonamide derivatives might have a different mechanism of action.

Besides the main mechanism of antifungal action, other mechanisms were described for azoles. In a previous work of our group, the inhibition of *C. albicans* and *C. krusei* biofilms by these 5-aminoimidazole-4-carbohydrazonamide derivatives was reported [17]. For miconazole, ROS production and effect against fungal biofilms were associated with its antifungal activity [5,12,21]. The interference of AA with compounds antifungal activity and biofilm formation was described [22,23,24]. AA strongly reacts with radicals instead of nonradical species, being AA scavenging potential represented by the hydroperoxyl radical/superoxide reactions [25]. In the present work we tried to evaluate if ROS production could be, at least in part, responsible for the antifungal effect of 2h, 2k and 2l. The results obtained when cells were treated with 5-aminoimidazole-4-carbohydrazonamide derivatives in the presence of AA (MIC values and ROS content) demonstrated the role of oxidative stress in the antifungal effect of compounds tested. Levels of 5 mM AA protected *C. albicans* and *C. krusei* from 2h, 2k and 2l toxicity.

Azoles can also act on mitochondria activity with the capacity to affect the mitochondrial respiratory chain and can be seen as potential cell growth inhibitors. In most eukaryotic cells, including fungi, mitochondria play several important functions such as generation and regulation of ROS, calcium (Ca^2+^) homeostasis and regulation of apoptosis, among other metabolic processes [26,27]. MTT is used to measure mitochondrial viability and function as it is cleaved in active mitochondria, producing a blue formazan product, and MTT-assay was used in this work to test the interference of 5-aminoimidazole-4-carbohydrazonamide derivatives in mitochondrial respiration of *C. albicans* and *C. krusei* [28]. The mitochondrial respiratory chain is an important internal source of superoxide radicals (O_2_^•−^) leading to yeast cell damage [29,30]. However, *Candida* sp. have superoxide dismutase (SOD), an enzyme that eliminates superoxide anions by converting them to H_2_O_2_, which is less toxic to cells [29]. It was also reported that *C. albicans* revealed an unusual SOD activity [31], processing six different types of SOD [29]. Additionally, Ramírez-Quijas and co-workers (2015) had already reported that among *Candida* species, *C. krusei* was the most resistant to H_2_O_2_, while *C. albicans* was the most resistant to superoxide anions [32]. These considerations might, in part, explain the mitochondrial activity/viability results here reported, which showed that *C. krusei* was significantly affected by 5-aminoimidazole-4-carbohydrazonamides treatment when compared to control, while *C. albicans* was less affected.

In yeast, ROS, including singlet oxygen, hydrogen peroxide and hydroxyl radicals, are metabolic byproducts from endogenous or exogenous sources. ROS commonly exist in the cell in balance with antioxidants, however the disruption of this balance due to an overproduction of ROS is related to marked cellular damage known as oxidative stress. In fact, several classes of antifungals, including fluconazole, have been reported as inducers of programmed cell death through ROS generation [33]. Accordingly, the results obtained also support a potential involvement of ROS generation in the antifungal activity of the 5-aminoimidazole-4-carbohydrazonamide derivatives tested.

When using H_2_O_2_ as positive control for ROS detection, higher concentrations were detected for *C. krusei* than for *C. albicans* compared to the control. *Candida albicans* have enzymes such as catalase and glutathione-peroxidase that are responsible for the quick degradation of H_2_O_2_ to H_2_O, making *C. albicans* one of the species of *Candida* genus most resistant to oxidative stress [34,35].

Antifungals can also affect the germ tube production and consequently interfere with the adhesion of yeasts to host cells [36,37]. *Candida albicans* is the most common pathogenic species of *Candida* genus because of its several virulence factors, which include the germ tubes formation [9], and some authors refer to the possibility of treating mycosis only by inhibition of *C. albicans* germ tube formation, due to its importance as virulence mechanism [38]. Compound 2h, although the less active (MIC 32–64 and MLC>128), was able to significantly inhibit *C. albicans* dimorphic transition, either in ATCC and azoles resistant strain H37, at a concentration of 32 µg/mL. This effect can potentiate the compound activity against *C. albicans*, by inhibition of this important virulence mechanism in this common species. By disturbing the yeast capacity to produce the germ tubes, fungistatic compounds reduce the yeasts adhesion to host cells, decreasing the evolution of the infection [36]. For 2k (MIC 32 and MLC 64) and 2l (MIC 16 and MLC 32), more active compounds than 2h, no significant inhibition of germ tube formation was observed, like for fluconazole, even for the concentrations of 2MIC. Several pathways have been described as being involved in the filamentation process of *C. albicans*, including NADH-dehydrogenase complex I [39]. Our results suggest that the mechanism underlying the inhibition of *C. albicans* filamentation by compound 2h was not mediated by the inhibition of the yeast NADH-dehydrogenase since this compound did not interfere with mitochondrial activity, as determined by the mitochondrial dehydrogenase activity (MTT) assay. On the other hand, although compound 2k significantly inhibited the mitochondrial activity of *C. albicans* for concentrations of 2MIC, no significant inhibition of the germ tube formation was observed for that concentration.

Combination therapy could be useful for the treatment of fungal infections, especially those caused by drug-resistant fungi. Concerning the possibility of a synergic effect with known antifungal agents, the association of the 5-aminoimidazole-4-carbohydrazonamide derivatives with fluconazole and amphotericin B did not result in an increase of activity for any of the compounds tested (2h, 2k or 2l). However, considering that fluconazole exhibits activity in *C. albicans* and is widely used for the treatment of candidosis by this species, its association with a compound such as 2h, which at concentrations lower than the MIC was able to add an inhibiting effect on filamentation, might help to better and more quickly solve the situation.

## 4. Materials and Methods

### 4.1. Standards and Reagents

Dimethyl sulfoxide (DMSO), sodium chloride (NaCl), 3-(*N*-morpholino) propanesulfonic acid (MOPS), *N*-acetylglucosamine, thiazolyl blue tetrazolium bromide (MTT), ergosterol, amphotericin B and fluconazole were purchased from Sigma-Aldrich (St. Louis, MO, USA). Proline was purchased from Fluka-Sigma-Aldrich (St. Louis, MO, USA). Ethanol and potassium hydroxide (KOH) were purchased from Panreac (Barcelona, Spain). HPLC-grade dichloromethane, *n*-heptane and ascorbic acid (AA) were purchased from Merck (Darmstadt, Germany). Sabouraud dextrose agar (SDA) and Sabouraud dextrose broth (SDB) were purchased from Bio-Mèrieux (Marcy L’Etoile, France). RPMI-1640 broth medium (with L-glutamine, without bicarbonate and with the pH indicator phenol red) was purchased from Biochrom AG (Berlin, Germany). Yeast nitrogen base was purchased from Difco (New Jersey, USA). Phosphate buffered saline (PBS) was purchased from Fisher Reagent (Geel, Belgium). Hydrogen peroxide (H_2_O_2_) was purchased from VWR (Fontenay-sous-Bois, France). 2’,7’-Dichlorodihydrofluorescein diacetate (H2DCFDA) was purchased from ThermoFisher Scientific (Life Technologies, Porto, Portugal).

### 4.2. Compounds

5-Aminoimidazole-4-carbohydrazonamide derivatives were synthesized by our group at Department of Chemistry of University of Minho, according to Ribeiro et al. (2014) [16]. Stock solutions of the compounds were prepared in DMSO and kept at −20 °C, and diluted in fresh culture medium just prior to the assays.

### 4.3. Fungal Organisms

Five *Candida* strains were used in this work: two *Candida* reference strains (*C. albicans* ATCC 10231 and *C. krusei* ATCC 6258) and three clinical strains kindly provided by Cidália Pina Vaz from CHSJ: *C. albicans* M1 (vulvovaginal recurrent candidiasis), *C. albicans* H37 (bronchoalveolar lavage fluid) and *C. dubliniensis* CD1 (blood). *Candida* strains were selected for the assays accordingly to the goals to be achieved. All microorganisms were kept in SDB plus glycerol (20%) at -80 °C. Before each test, a 24 h sub-culture in SDA was prepared to achieve optimal growth conditions and purity.

### 4.4. Susceptibility Tests

Minimal inhibitory concentrations (MIC) were obtained with recourse to the broth microdilution test of reference document M27A-3 from Clinical and Laboratory Standard Institute (CLSI) [40]. In a summarized way, the yeasts from 24 h cultures on SDA were suspended in RPMI-1640 broth buffered with MOPS (pH 7.0) to get 10^3^ colony forming units (CFU)/mL. Two-fold serial dilutions of imidazole derivatives solved in DMSO were prepared in RPMI-1640 broth, starting from 128 to 0.5 µg/mL. The same range of imidazoles concentrations were tested combined with AA (5 mM). The solutions and cell suspensions were distributed (100 μL of each one) into sterile 96-well plates. The plates were incubated aerobically in a humid atmosphere, at 35 °C during 48 h and growth was indicated by turbidity. MIC was the lowest concentration inhibiting 100% growth when compared to compound-free control. Controls: quality, using *C. krusei* ATCC 6258 and performed with fluconazole; sterility, with RPMI-1640 medium; and growth, with RPMI-1640 medium plus DMSO (1.0%; *v*/*v*). From plates used for testing MIC, yeasts suspension (20 μL) from wells without visible growth were transferred to SDA plates and these were incubated at 35 °C and checked for growth after 24 h, in order to evaluate the minimum lethal concentration (MLC), defined as the lowest concentration showing a growth inhibition of 100%. All the experiments were repeated independently at least three times. A range of values is presented when different results were obtained.

### 4.5. Cell Membrane Ergosterol

The effect on inhibition of ergosterol synthesis in *C. albicans* ATCC 10231 and *C. krusei* ATCC 6258 was determined according to Pinto et al. (2011) with minor modifications [41]. Briefly, 10 µL of yeasts cell suspensions, at 0.5 McFarland, were inoculated in 5 mL of RPMI-1640 medium containing different concentrations of the imidazole derivatives. A control with fluconazole was included, as well as a control without compounds. Cultures were incubated at 35 °C with shaking during 48 h. After incubation yeast cells were collected by centrifugation (4000 rpm, 5 min), twice washed with distilled water, dried and weighted. The intracellular sterols were extracted by saponification [42]. Cell pellets in sterile borosilicate glass tubes with 25% alcoholic KOH solution (3 mL) were vigorously agitated in a vortex. Cell suspensions were placed in a water bath (85 °C) for 60 min and cooled at room temperature. Afterwards sterols extraction was performed by adding a mixture of sterile distilled water (1 mL) and *n*-heptane (3 mL) followed by vigorous vortex agitation during 5 min. The solvent of the organic phase was evaporated to dryness using nitrogen stream, after transference to glass tubes. The extracted sterols were dissolved in 0.5 mL of dichloromethane. Prior to HPLC-DAD analysis the samples were filtered (0.2 µm) and ergosterol was used as standard [41]. The experiments were repeated independently two times.

### 4.6. Cell Metabolic Viability

The effect of 5-aminoimidazole-4-carboxamidrazones derivatives on yeasts metabolic viability was evaluated through mitochondrial dehydrogenase activity, using an MTT assay based on the method described by Lopes et al. (2013) with some modifications [9]. Briefly, suspensions of yeasts (*C. albicans* ATCC 10231 and *C. krusei* ATCC 6258) with final cell density of 0.5–2.5 × 10^3^ CFU/mL were prepared in RPMI 1640 medium. These work suspensions were placed into micro tubs (1 mL), incubated for 18–24 h, at 37 °C and then were centrifuged at 10,000 rpm during 5 min. To the cell pellet obtained, imidazole derivatives or fluconazole were added to obtain the desired concentrations, and incubated by 1 h at 37 °C. Treated cells were centrifuged (10,000 rpm for 5 min) and exposed to 500 μL of an MTT solution prepared in RPMI (0.5 mg/mL) for 30 min at 37 °C. Formazan products were solubilized with 300 μL of DMSO and the purple color intensity evaluated at 510 nm using a spectrophotometer (Synergy HT plate reader; BioTeck Instruments, Highland Park, IL, USA).

### 4.7. Total Intracellular ROS Measurement

*Candida albicans* ATCC 10231 and *C. krusei* ATCC 6258 cell suspensions in RPMI-1640 medium were prepared from recent cultures on SDA and incubated in a water bath, at 37 °C, with agitation, for 18–24 h. Suspensions were centrifuged, cells washed and resuspended in sterile PBS in order to obtain a final density of approximately 10^6^ cells/mL. Imidazole derivatives and AA stocks were freshly prepared in DMSO. Stocks solutions were then dissolved in PBS in order to achieve the different desired concentrations (4MIC, 2MIC and MIC values for imidazoles and 5mM for AA) after addition to yeast suspensions. The effect of compounds was tested either alone or combined with 5 mM of AA. Solutions of DMSO and H_2_O_2_ were prepared in PBS to achieve final concentrations of 0.5% and 1 mM, respectively, after addition to yeast suspensions. Compounds were added to cell suspension into micro tubs and incubated at 37 °C in a water bath with agitation, for 6 h. A control of cells suspended only in PBS was also included in the assay. After the exposure time, cells were centrifuged at 15,000 rpm for 10 min and pellets were resuspended in 500 µL PBS. Analysis of ROS generation was performed by flow citometry using 8 µL of 2’,7’-dichlorodihydrofluorescein diacetate (H2DCFDA), for 30 min at 37 °C. The AccuriTM C6 flow cytometer and the BD Accuri C6 software (BD Biosciences) were used.

### 4.8. Germ Tube Inhibition Assay

*C. albicans* strains, ATCC 10231 and clinical isolate H37, were used to evaluate the 5-aminoimidazole-4-carboxamidrazone derivatives effect on the yeast-mycelium transition. Overnight cultures in SDA were used to prepare yeast cell suspensions (1.0 ± 0.2 × 10^6^ CFU/mL) in NYP medium (*N*-acetylglucosamine (10^−3^ mol/L), Yeast Nitrogen Base (3.35 g/L), proline (10^−3^ mol/L), NaCl (4.5 g/L); pH 6.7 ± 0.1) [43]. Suspensions were distributed into glass test tubes (final volume of 990 μL) to which 10 μL of imidazole derivatives or fluconazole solutions were added to obtain concentrations of 2MIC, MIC and 1/2MIC. After a 3 h incubation at 37 °C, 100 cells from treated and untreated samples were observed under a light microscope. Germ tube positive cells were considered when the germinating tube was at least as long as the diameter of the blastospore and the percentage of germinating cells was calculated for three independent experiments, being the results expressed as average ± standard error of the mean (SEM).

### 4.9. Checkboard Microdilution Assay

The checkerboard method remains the furthermost popular for evaluating drug interactions between antifungals, although with some limitations [44]. The micromethod, using a 96 well plate, was used to evaluate the MICs for the different imidazole derivatives, alone and in combination with antifungals fluconazole and amphotericin B, for *C. albicans* ATCC 10231 and *C. krusei* ATCC 6258. The plates were incubated at 35 °C and the results of the MIC were read after 48 h. The fractional inhibitory concentration index was calculated for the compound combinations, meaning “synergism” a FICI < 0.5, “antagonism” a FICI > 4 and “without interaction” a FICI between 0.5 and 4 [44]. The results were obtained from tests repeated three times.

### 4.10. Statistical Analysis

Data analyses were carried out in GraphPad Prism Software version 8.4.0. One-way or two-way analysis of variance (ANOVA) was performed followed by Dunnett’s test. Levels of statistical significance *p* < 0.05, *p* < 0.01 and *p* < 0.001 were used.

## 5. Conclusions

ROS production seems to be implicated in the antifungal activity of 5-aminoimidazole-4-carbohydrazonamide derivatives tested. The higher antifungal activity observed against *C. krusei* comparing to *C. albicans*, reflected in MIC values obtained for the compounds, may be related to the unequal sensitivity of the two *Candida* species to the different ROS. Future work will study the chemical reactivity of the compounds to establish the influence of structure of the molecule in its capacity to generate ROS.

Although no synergism was verified for the compound 2h with fluconazole, the association with this antifungal drug might be valuable because of the inhibition of the dimorphic transition, a virulence mechanism of *C. albicans*.

## Figures and Tables

**Table 1:** Antifungal activity (minimal inhibitory concentration (MIC) and minimal lethal concentration (MLC)) of 5-aminoimidazole-4-carbohydrazonamide derivatives against *Candida* strains.

Compounds	*Candida* Strains	MIC (µg/mL)	MLC (µg/mL)
2h	*C. albicans* ATCC	32–64	>128
	*C. albicans* H37	64	>128
	*C. albicans* M1	64	>128
	*C. dubliniensis* CD1	64	>128
	*C. krusei* ATCC	4–8	8
2k	*C. albicans* ATCC	32	64
	*C. albicans* H37	32	64
	*C. albicans* M1	16	64
	*C. dubliniensis* CD1	16	32
	*C. krusei* ATCC	8	≥32
2l	*C. albicans* ATCC	16	32
	*C. albicans* H37	16	≥32
	*C. albicans* M1	8	16
	*C. dubliniensis* CD1	8	≥16
	*C. krusei* ATCC	4	4
Fluconazole	*C. albicans* ATCC	1	>128
	*C. albicans* H37	64	>128
	*C. albicans* M1	2	128
	*C. dubliniensis* CD1	1	>128
	*C. krusei* ATCC	64	64–128

**Table 2:** Influence of ascorbic acid on antifungal activity of 5-aminoimidazole-4-carbohydrazonamide derivatives against *Candida albicans* ATCC 10231 and *Candida krusei* ATCC 6258.

Compounds/ Species	MIC (µg/mL)					
	2h	2h + AA	2k	2k + AA	2l	2l + AA
*C. albicans*	32–64	>128	32	>128	16	>128
*C. krusei*	4–8	>32	8	>32	4	>32

MIC, minimal inhibitory concentration; AA, ascorbic acid (5 mM).
